# Benefits of a mentoring support program for individuals with an eating disorder: a proof of concept pilot program

**DOI:** 10.1186/s13104-017-3026-6

**Published:** 2017-12-07

**Authors:** Lucie M. Ramjan, Phillipa Hay, Sarah Fogarty

**Affiliations:** 1 School of Nursing and Midwifery, Centre for Applied Nursing Research, Ingham Institute of Applied Medical Research, Western Sydney University, Locked Bag 1797, Penrith, NSW 2751 Australia; 2 School of Medicine and Centre for Health Research, Western Sydney University, Locked Bag 1797, Penrith, NSW 2751 Australia; 3 School of Nursing and Midwifery, Western Sydney University, Locked Bag 1797, Penrith, NSW 2751 Australia

**Keywords:** Mentoring support, Eating disorders, Anorexia nervosa

## Abstract

**Objective:**

The aim of this proof-of-concept pilot study was to assess the usefulness of a mentoring support program with a goal to improve hope for recovery in people with an eating disorder.

**Results:**

Significant improvements (p ≤ 0.05) in hope for recovery were found for the mentees in the following domains: social relationships (p = 0.027), romantic relationships (p = 0.032), family life (p = 0.047), work (0.003) and overall scores (p = 0.003). There were no significant findings for any of the areas for the mentors. Despite this, mentoring programs that focus on improving hope may provide a valuable adjunct support for those in treatment for an eating disorder.

**Electronic supplementary material:**

The online version of this article (10.1186/s13104-017-3026-6) contains supplementary material, which is available to authorized users.

## Introduction

Mentoring in the context of eating disorders, is a relationship between “a person with a lived experience of an eating disorder who has recovered” (mentor) and a person with an eating disorder (mentee) [1, 2]. Mentoring aims to improve self-esteem and body image [2–4] however as an adjunct to established treatments has had limited attention [2]. We know of no trials which have investigated the benefits of mentoring as a positive adjunct to improving hope [2, 4]. Hope has been shown to be important in recovery from an eating disorder [5–10] and linked to an enhanced therapeutic alliance, improved treatment compliance and behavioral change [10, 11]. Hope also provides a buffer against stress and assists in coping during difficulty and suffering [12]. Thus a mentoring program for individuals with an eating disorder may provide support that promotes hope for recovery and or a better quality of life. The primary aim of this “proof of concept” trial was to assess the impact of a mentoring support program on instilling hope in individuals with an eating disorder.

## Main text

### Method

#### Population, sample and procedure

**Fig. 1:**
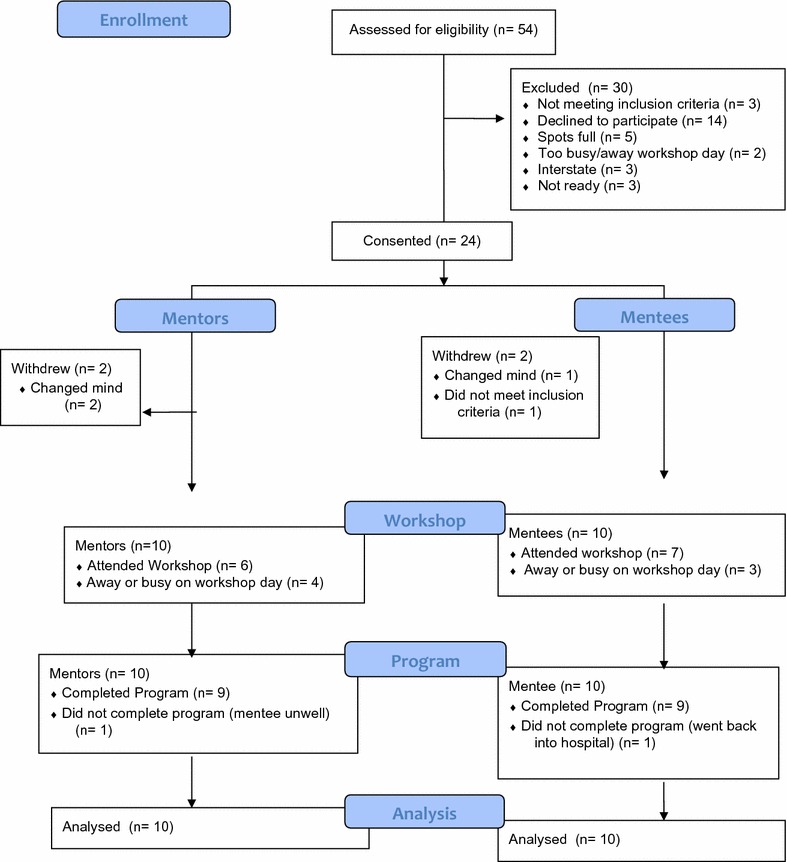
CONSORT flow diagram

The pilot study was advertised, in eating disorder treatment facilities and online through relevant websites. Fifty-four individuals responded, see Fig. 1. The mentee sample (N = 10 females) were women with a mixture of eating disorders; five with anorexia nervosa (AN), two with severe and enduring AN (SEAN), and one each with binge eating disorder (BED), bulimia nervosa (BN) and other specified or unspecified feeding or eating disorders (OS/UFED), aged between 20 and 42 years (mean 29.2/SD 8.2). The mentor sample (N = 10 females) were women recovered from AN (some of whom also previously had BN), aged between 23 and 52 (mean 28.9/SD 8.2) who had on average 5 years recovery from the eating disorder. Recovery was self-reported by the mentors and verified by interview with a psychiatrist (author PH). The psychiatrist assessed recovery and the mentors’ suitability [including determining that the mentor had appropriate psychological support(s) in place]. Mentors were excluded if assessed as unsuitable. All mentors and mentees had to be ≥ 18 years of age. Mentees had to have an eating disorder. Mentors had to be recovered. The study was conducted in Sydney, Australia and all mentees were from Sydney. Nine of the mentors were from Sydney and one was from the USA. One mentee specifically requested an online relationship due to severe social anxiety and was paired with the mentor from the USA. The University of Western Sydney Human Research Ethics Committee approved the study (H10825).

#### Mentoring program

The mentoring program was an adjunct to individuals’ eating disorder treatment. As no mentoring programs have assessed the promotion of hope, a proof-of-concept (POC) study was considered. Proof-of-concept studies are considered when the objective of the study is to obtain an initial evaluation of the potential benefit of a treatment or program [13]. Participatory action research (PAR) principles founded program development. PAR “is a form of action research that focuses on the participatory aspect of research in action” [14]. PAR allows participants the flexibility to determine aspects of the program that suit their needs within a framework. Whilst aspects of the program were flexible e.g. when and how communication occurred, there were non-negotiables for all mentoring dyads which included: the program length, minimum of three face-to-face meetings, average time spent with mentor/mentee (approx. 1 h/week), reiteration that the mentor was not a counselor and crisis care and risk management plans, including mandatory reporting of child abuse and risk of self-harm. In addition, the investigators (SF/LR) monitored mentors and mentees regularly. See Nicholls et al. [14] for details of a similar mentoring program. The program ran for 13 weeks. There were ten mentor–mentee pairs.

### Measures

There were two types of measures used in the study: (1) those to assess the benefits of the program and; (2) those to monitor the health and well-being of the participants during the study.

#### The benefits of the program

The primary outcome measure was hope. Hope was measured using the validated Domain Specific Hope Scale [15]. This scale is a 50 item, self-report instrument for measuring an individual’s level of dispositional hope in relation to 6 life areas—social, academic, family, romance/relationships, work/occupation, and leisure activities. The higher the score the greater the level of hope. It has robust psychometrics [15].

The secondary outcome measures included quality of life, distress and the mentoring relationship. Quality of life was assessed with the validated self-report questionnaires; SF-12 [16] and the Eating Disorder Quality of Life Scale (EDQoL) [17]. The SF-12 is a 12-item self-report questionnaire that measures functional health and well-being using two subscales; the physical component summary and the mental component summary [16]. The EDQoL scale is a 25-item self-report measure assessing the degree to which an individual feels their eating disorder affects their quality of life [17]. There are four subscales: psychological, physical/cognitive, work/school and financial.

Distress was assessed with the Kessler Psychological Distress Scale (K10) [18]. The K10 is a 10-item self-report questionnaire that yields a global measure of distress based on questions about anxiety and depressive symptoms. It has robust psychometrics [18].

The perception of the mentoring relationship was assessed using the validated match characteristic questionnaire (MCQ) [19]. The MCQ is a 29 item, self-report instrument for measuring positive and negative perceptions of the mentoring relationship, the valuation of different purposes in the match, and the effects of external influences on the match [19].

These measures were assessed at baseline (before the program started) and at completion of the program except for the MCQ, which was assessed post program only.

#### The health and wellbeing of participants

The following were used to monitor the participants well-being during the study: 12-item short form survey (SF-12) [16] and the Eating Disorder Quality of Life Scale (EDQoL) [17], Kessler Psychological Distress Scale (K10) [18], eating concerns with the short version eating disorder examination (EDE-Q) [20] and the perception of the mentoring relationship with Global Mentoring Relationship Questionnaire Scale (GMeRQS) [21]. Individuals completed assessments mid program (at 7 weeks) and post program except for the GMeRQS which was completed at weeks 3 and 9 of the program to monitor stages of the mentoring relationship. Fortnightly logbooks qualitatively measured participants’ health and the mentoring relationship by providing feedback on issues.

### Analysis

All outcomes (except for the MCQ) assessing the benefit of the program were analysed using a paired *t* test comparing baseline and the post treatment results using Microsoft Excel. Standard scoring methods were applied to the validated questionnaires.

The MCQ was scored using the tool available from http://www.mentoringevaluation.com. This scores each of the domains. The Australian scoring tool was used and provides a population average for comparison against the mentors only. As the mentees could not be compared to any means there were no comparisons. The MCQ scoring was done only post program.

Intent to treat was applied for the incomplete questionnaires by carrying baseline over to post-program scores. Outcome measures to assess the health and well-being of the participants were subjected to inferential statistical analysis.

### Results

The dyad who corresponded via email only discontinued the program 2 weeks after commencement, as the mentee returned to hospital (eating disorder related). This couple did not complete any further questionnaires. In addition one mentee did not complete any of the post program questionnaires.

#### Presentation of the quantitative outcomes

Medians and standard deviations for the data for assessing benefit are reported (see Tables 1 and 2). Significant results (p ≤ 0.05) were found between pre and post program scores for the mentees in increasing hope overall (p = 0.003) and in the following hope domains: social relationships (p = 0.027), Romantic relationships (p = 0.032), family life (p = 0.047), and Work (0.003). There were no other significant results for the other outcome measures; however, in all outcome measures, except for the EDQoL Psychological domain, mentees outcome measures improved or were stable on average.

**Table 1:** Pre and post program values and results for mentees

	Baseline (median/SD)	Post-program (median/SD)	Scoring direction	p values
Mentees (n = 10)				
Domain Hope Scale				
Social relationships	37.6 (15.8)	42.5 (17.9)	Higher = greater hope	0.027*
Academics	48.7 (12.0)	51.4 (12.4)		0.145
Romantic relationships	22.9 (13.7)	27.6 (15.9)		0.032*
Family life	40.0 (13.5)	43.7 (13.4)		0.047*
Work	46.1 (16.9)	51.8 (14.49)		0.003*
Leisure activities	39.7 (11.9)	44.1 (14.0)		0.098
Total	235.0 (64.3)	261.9 (64.9)		0.003*
SF-12 quality of life				
Physical	47.8 (12.3)	49.3 (12.8)	Higher = greater quality of life	0.492
Emotional	30.7 (6.85)	30 8 (9.67)		0.977
EDQoL				
Psychological	24.3 (8.74)	26.7 (5.08)	Lower = greater quality of life	0.252
Physical	11.6 (5.74)	11.3 (6.77)		0.782
Financial	8.10 (6.69)	5.80 (6.65)		0.285
Work/school	4.40 (3.95)	3.30 (4.08)		0.075
Total	48.4 (18.5)	47.1 (18.9)		0.381
K10				
	32.0 (8.0)	31.4 (10.1)	Higher = greater distress/anxiety	0.698

* Denotes significance at p < 0.05

**Table 2:** Pre and post program values and results for mentors

	Baseline (median/SD)	Post-program (median/SD)	Scoring direction	p values
Mentors (n = 10)				
Domain Hope Scale				
Social relationships	54.5 (6.38)	42.5 (17.9)	Higher = greater hope	0.431
Academics	54.7 (5.33)	51.4 (12.4)		0.434
Romantic relationships	47.9 (7.96)	27.6 (15.9)		0.198
Family life	57.7 (4.81)	43.7 (13.4)		0.300
Work	57.0 (4.85)	51.8 (14.49)		0.528
Leisure activities	55.5 (5.50)	44.1 (14.0)		0.663
Total	327.0 (24.7)	261.1 (64.9)		0.387
SF-12 quality of life				
Physical	54.1 (4.71)	54.4 (6.94)	Higher = greater quality of life	0.706
Emotional	51.8 (7.27)	50.2 (6.55)		0.435
EDQoL				
Psychological	1.3 (1.95)	1.6 (2.50)	Lower = greater quality of life	0.754
Physical	0.0 (0.0)	0.0 (0.0)		NA
Financial	0.0 (0.0)	0.10 (0.32)		0.343
Work/school	0.0 (0.0)	0.0 (0.0)		NA
Total	1.3 (1.95)	1.7 (2.54)		0.662
K10				
	13.1 (1.92)	13.3 (1.85)	Higher = greater distress/anxiety	0.661

*  Denotes significance at p < 0.05

Population averages were only available for the mentors for the MCQ and the mentors were generally similar to the population norms (see Additional file 1). The mentors did not feel as close and felt more distance between themselves and the mentees than the population norm. The mentors perceived the mentees needed support (both academic and non-academic), which was greater than the population norm. The mentors also valued talking and sharing more than the population norm.

There were no significant results for the mentors. The mentors’ outcome measures remained stable from pre to post program completion.

### Discussion

There was a significant increase in hope for the mentees from baseline to post program. The authors found no mentoring and eating disorder trials that assessed hope however there is considerable qualitative research that shows that hope is an important aspect in the recovery process from an eating disorder [5–10]. Previous research shows hope has been linked to improved treatment compliance and has improved the therapeutic alliance in treatment for those with severe and enduring AN [10]. The focus of this program was not to improve treatment compliance but future studies may want to consider using treatment compliance as an outcome measure. Recovery from an eating disorder is difficult [6] and any adjunct treatment that supports recovery is valuable.

While improvement in hope was seen for the mentees as a group, specifically the two participants with severe and enduring AN had small increases in hope from baseline to post program. Hope is an important factor in the recovery process from longstanding AN. These individuals are often resistant to traditional treatments with high levels of impairment in most aspects of life with poor outcomes [6]. Mentoring programs that focus on improving hope may provide valuable adjunct support for those in treatment for an eating disorder including those with SEAN. Further research could specifically investigate the use of a mentoring program for individuals with SEAN.

One of the concerns of a mentoring program for individuals with an eating disorder is maintaining safety. The study results show that the mentors remained stable during the program and although non-significant in all aspects, except for reported hope domains, the mentees, on average, improved or were stable in all study outcomes, except the EDQoL psychological domain for which there was a small decrease. The reason for the decrease in the psychological EDQoL domain is unknown. The mid-point EDQoL results for mentees also showed a small average rise so it seems unlikely that the end of the program influenced quality of life. Examination of the individual answers to the psychological domain questions did not indicate a specific decrease in any one aspect of psychological QoL. The program can be viewed as ‘safe’ for both mentors and mentees.

Interest exceeded expectations and resources indicating that individuals with an eating disorder are interested in a mentoring program. Given this, it is feasible that a larger study could recruit the mentee participant numbers needed. Further given the increase of hope seen and the promising results that indicate program safety, future efficacy randomised controlled trials are warranted.

### Conclusion

The mentoring support program showed promise in improving hope. The program appears ‘safe’ for both mentors and mentees. Further research involving a larger sample size is needed to replicate these findings.

### Limitations

It is important to note that the program had no comparator intervention so whilst the results are promising it is not within the scope of this study to determine its efficacy.

## Additional file

**Additional file 1.** MCQ post-program values and results for mentees and mentors. MCQ post-program results for mentors compared to population norms.

## Abbreviations

AN: Anorexia nervosa
SEAN: severe and enduring anorexia nervosa
BED: binge eating disorder
BN: bulimia nervosa
OS/UFED: other specified or unspecified feeding or eating disorder
POC: proof-of-concept
PAR: participatory action research
EDQoL: Eating Disorder Quality of Life Scale
K10: Kessler Psychological Distress Scale
MCQ: match characteristic questionnaire
SF-12: 12-item short form survey
EDE-Q: eating disorders examination questionnaire
GMeRQS: Global Mentoring Relationship Questionnaire Scale

## References

[1] Collins Dictionary of the English Language. In: Wilkes GAE, editor. Collins Dictionary of the English Language. Sydney: Collins; 1979.

[2] Perez M, Van Diest AK, Cutts S (2014). Preliminaray examination of a mentor-based program for eating disorders. J Eat Dis.

[3] McVey G, Kirsh G, Maker D, Walker K, Mullane J, Laliberte M (2010). Promoting positive body image among university students: a collaborative pilot study. Body Image.

[4] Lippi DE. The impact of the mentoring relationship upon women in the process of recovering from eating disorders. Bell & Howell Information and Learning Company: Temple University; 2000.

[5] Fogarty S, Ramjan LM, Hay P (2016). A systematic review and meta-synthesis of the effects and experience of mentoring in eating disorders and disordered eating. Eat Behav.

[6] Dawson L, Rhodes P, Touyz S (2014). “Doing the Impossible”: the process of recovery from chronic anorexia nervosa. Qual Health Res.

[7] Lindgren B-M, Enmark A, Bohman A, Lundström M (2015). A qualitative study of young women’s experiences of recovery from bulimia nervosa. J Adv Nurs.

[8] Hay PJ, Cho K (2013). A qualitative exploration of influences on the process of recovery from personal written accounts of people with anorexia nervosa. Women Health.

[9] Las Hayas C, Padierna J, Muñoz P, Aguirre M, Gómez Del Barrio A, Beato-Fernández L (2016). Resilience in eating disorders: a qualitative study. Women Health.

[10] Stiles-Shields C, Touyz S, Hay P, Lacey H, Crosby R, Rieger E (2013). Therapeutic alliance in two treatments for adults with severe and enduring anorexia nervosa. Int J Eat Disord.

[11] Castonguay L, Pincus A, Agras W, Hines C (1998). The role of emotion in group cognitive-behavioral therapy for binge eating disorder: when things have to feel worse before they get better. Psychother Res.

[12] Miller J, Miller J (1999). Inspiring hope. Coping and chronic illness: overcoming powerlessness.

[13] Gewandter JS, Dworkin RH, Turk DC, McDermott MP, Baron R, Gastonguay MR (2014). Research designs for proof-of-concept chronic pain clinical trials: IMMPACT recommendations. Pain.

[14] Nicholls D, Fogarty S, Hay P, Ramjan LM (2016). Participatory action research for women with anorexia nervosa. Nurse Res.

[15] Simpson S (1999). Validation of the Domain Specific Hope Scale: exploring hope in life domains.

[16] Ware JJ, Kosinski M, Keller S (1996). A 12 item short form health survey: construction of scales and preliminary tests of reliability and validity. Med Care.

[17] Engel SG, Wittrock DA, Crosby RD, Wonderlich SA, Mitchell JE, Kolotkin RL (2005). Development and psychometric validation of an eating disorder-specific health-related quality of life instrument. Int J Eat Disord.

[18] Kessler RC, Andrews G, Colpe L, Hiripi E, Mroczek D, Normand S (2002). Short screening scales to monitor population prevalences and trends in non-specific psychological distress. Psychol Med.

[19] Karcher MJ, Nakkula MJ, Harris J (2005). Developmental mentoring match characteristics: correspondence between mentors’ and mentees’ assessments of relationship quality.

[20] Cooper Z, Fairburn CG (1987). The eating disorder examination: a semi-structured interview for the assessment of the specific psychopathology of eating disorders. Int J Eat Disord.

[21] Ferro A, DeWit D, Wells S, Lipman E (2013). An evaluation of the measurement properties of the Mentor Self-Efficacy Scale among participants in Big Brothers Big Sisters of Canada Community Mentoring Programs. Int J Evid Based Coach Mentor.

