# Supplementary material for: Benefits of a mentoring support program for individuals with an eating disorder: a proof of concept pilot program
Source: BMC Res Notes. 2017 Dec 7;10:709. doi: 10.1186/s13104-017-3026-6 (PMC5719736; doi:10.1186/s13104-017-3026-6)
Supplement: Supplementary file 1 — Additional file 1. MCQ post-program values and results for mentees and mentors. MCQ post-program results for mentors compared to population norms. [file 13104_2017_3026_MOESM1_ESM.docx]

**Additional file 1**

*MCQ post program values and results for mentees and mentors.*

| **Mentees (n=10)** | | | | |
| --- | --- | --- | --- | --- |
| MCQ | | | | |
|  | Score Meaning | Post-Program  (Median/IQR) | Scoring Direction | Population Average # |
| Compatibility Score | How generally compatible does the mentee feel with the mentor? | 69.2 (24.5) | Higher scores = greater satisfaction | *Not available* |
| Handle Issues Score | How well prepared does the mentee feel the mentor is able to handle the mentee's issues? | 72.5 (28.2) | Higher scores = greater satisfaction | *Not available* |
| Closeness Score | How close does the mentee feel with the mentor? | 66.7 (17.3) | Higher scores = greater satisfaction | *Not available* |
| Discomfort Score | How awkward/distant does the mentee feel with the mentor? | 81.2 (20.1) | Higher scores = greater satisfaction | *Not available* |
| Competence Score | How competent does the mentee feel the mentor is? | 70.0 (17.9) | Higher scores = greater satisfaction | *Not available* |
| Satisfaction Score | How generally satisfied does the mentee feel with the match? | 61.3. (12.5) | Higher scores = greater satisfaction | *Not available* |
| Nonacademic Support-Seeking | How much does the mentee perceive the mentor provides non-academic support? | 52.7 (13.6) | Higher scores = greater satisfaction | *Not available* |
| Academic Support-Seeking | How much does the mentor perceive the mentor provides academic support? | 34.4 (27.9) | Higher scores = greater satisfaction | *Not available* |
| Fun Purpose Score | How much does the mentee value doing fun activities with the mentor? | 55.6 (18.3) | Higher scores = greater value for these activities | *Not available* |
| Sharing Purpose Score | How much does the mentee value talking/sharing with the mentor? | 63.9 (17.3) | Higher scores = greater value for these activities | *Not available* |
| Character Dev't Purpose Score | How much does the mentee value the mentor developing the mentee's character? | 47.8 (22.2) | Higher scores = greater value for these activities | *Not available* |
| Outlook Purpose Score | How much does the mentee value the mentor improving the mentee's outlook? | 64.4 (21.4) | Higher scores = greater value for these activities | *Not available* |
| Academics Purpose Score | How much does the mentee value the mentor improving the mentee's academics? | 61.7 (15.8) | Higher scores = greater value for these activities | *Not available* |
| Programmatic Support Score | How much does the mentee feel supported by the program? | 55.0 (23.8) | Higher scores = greater levels of support | *Not available* |
| Parental Engagement Score | How positively are the mentee's parents are engaged with the match? | 73.8 (17.7) | Higher scores = greater levels of support | *Not available* |
| Peer Support Score | How much support does the mentee get from peers (family/friends)? | 32.5 (29.6) | Higher scores = greater levels of support | *Not available* |
| Interference Score | How much do logistics and circumstances interfere with the match? | 71.3 (15.5) | Higher scores = lower levels of interference | *Not available* |
| **Mentors (n=10)** | | | |  |
| MCQ | | | |  |
|  | Score Meaning | Post-Program  (Median/IQR) | Scoring Direction | Population Average |
| Compatibility Score | How generally compatible does the mentor feel with the mentee? | 80.6 (16.6) | Higher scores = greater satisfaction | *77.0* |
| Handle Issues Score | How well prepared does the mentor feel the mentee is able to handle the mentee's issues? | 82.0 (16.3) | Higher scores = greater satisfaction | *85.1* |
| Closeness Score | How close does the mentor feel with the mentee? | 56.8 (17.8) | Higher scores = greater satisfaction | *64.8* |
| Discomfort Score | How awkward/distant does the mentor feel with the mentee? | 82.3 (9.40) | Higher scores = greater satisfaction | *75.9* |
| Competence Score | How competent does the mentor feel? | 76.0 (13.1) | Higher scores = greater satisfaction | *Not available* |
| Satisfaction Score | How generally satisfied does the mentor feel with the match? | 66.0 (10.7) | Higher scores = greater satisfaction | *69.9* |
| Nonacademic Support-Seeking | How much does the mentor perceive the mentee seeks non-academic support? | 56.9 (14.8) | Higher scores = greater satisfaction | *47.9* |
| Academic Support-Seeking | How much does the mentor perceive the mentor seeks academic support? | 53.3 (19.4) | Higher scores = greater satisfaction | *30.0* |
| Fun Purpose Score | How much does the mentor value doing fun activities with the mentee? | 63.0 (21.8) | Higher scores = greater value for these activities | *63.7* |
| Sharing Purpose Score | How much does the mentor value talking/sharing with the mentee? | 73.5 (13.6) | Higher scores = greater value for these activities | *54.8* |
| Character Dev't Purpose Score | How much does the mentor value developing the mentee's character? | 56.1 (14.1) | Higher scores = greater value for these activities | *66.3* |
| Outlook Purpose Score | How much does the mentor value improving the mentee's outlook? | 65.6 (16.7) | Higher scores = greater value for these activities | *64.3* |
| Academics Purpose Score | How much does the mentor value improving the mentee's academics? | 60.6 (16.1) | Higher scores = greater value for these activities | *60.7* |
| Programmatic Support Score | How much does the mentor feel supported by the program? | 65.2 (14.4) | Higher scores = greater levels of support | *59.4* |
| Parental Engagement Score | How positively are the mentee's parents are engaged with the match? | 76.0 (20.1) | Higher scores = greater levels of support | *Not available* |
| Peer Support Score | How much support does the mentee get from peers (family/friends)? | 56.7 (31.6) | Higher scores = greater levels of support | *Not available* |
| Interference Score | How much do logistics and circumstances interfere with the match? | 77.5 (17.5) | Higher scores = lower levels of interference | *83.6* |

# Population norms are not available for mentees or those with an eating disorder.
